# Dehydrated silk fibroin matrices as versatile delivery systems for extracellular vesicles

**DOI:** 10.3389/fbioe.2026.1873981

**Published:** 2026-07-07

**Authors:** Alp Sarisoy, Yong Xu, Stephan Rütten, Eva Miriam Buhl, Cecile Maire, Sandra Fuest, Ralf Smeets, Franz Lennard Ricklefs, Christian Apel

**Affiliations:** 1 Department of Biohybrid & Medical Textiles (BioTex), Institute of Applied Medical Engineering, Uniklinik RWTH Aachen, Aachen, Germany; 2 Electron Microscopy Facility, Institute of Pathology, Uniklinik RWTH Aachen, Aachen, Germany; 3 Department of Neurosurgery, University Medical Center Hamburg-Eppendorf, Hamburg, Germany; 4 Department of Oral and Maxillofacial Surgery, University Medical Center Hamburg-Eppendorf, Hamburg, Germany; 5 Department of Oral and Maxillofacial Surgery, Division of Regenerative Orofacial Medicine, University Medical Center Hamburg-Eppendorf, Hamburg, Germany

**Keywords:** controlled release, extracellular vesicle delivery systems, extracellular vesicles, films, nonwovens, silk fibroin, wound healing

## Abstract

Extracellular vesicles (EVs) have emerged as promising alternatives to cell-based therapies due to their low immunogenicity, ability to carry therapeutic cargo, and enhanced stability. However, rapid clearance from the bloodstream and phagocytic uptake by macrophages after systemic or local administration limit their therapeutic efficacy, highlighting the need for delivery systems that enable sustained and localized EV release. In this study, a natural protein silk fibroin (SF) was selected as a biomaterial carrier due to its tunable degradation kinetics and biocompatibility. SF was produced into nonwovens and films to investigate the effect of material structure on EV delivery in dehydrated SF matrices. A mild water vapor annealing approach was applied to tune structural stability and β-sheet formation while preserving EV integrity. Gingival fibroblast (GF)-derived EVs were successfully incorporated into nonwovens and films. EV-loaded nonwovens and films demonstrated distinct release characteristics over 14 days, with nonwovens providing a more controlled release and films showing delayed but accelerated release at later time points. EV morphology was preserved after release, and the EVs were notably internalized by human umbilical vein endothelial cells (HUVECs). Both EV-loaded materials enhanced the migration in a wound healing assay. These findings highlight the potential of dehydrated SF matrices as promising EV delivery systems for future regenerative medicine applications.

## 1 Introduction

Due to their cell-free nature, extracellular vesicles (EVs) demonstrated remarkable potential over cell-based therapies in regenerative medicine. They carry various therapeutic agents consisting of mRNAs, miRNAs, and proteins such as growth factors. In addition to their prolonged storage time, EVs have reduced immunogenicity and provide immunomodulatory properties (Mulcahy et al., 2014; Herrmann et al., 2021). Their regenerative properties have been shown in various applications, including drug delivery, wound healing and cancer therapy (Lee et al., 2014; Xian et al., 2018; Harrell et al., 2019; Liu et al., 2021). However, despite their outstanding features, the clinical translation of EV-based therapies remains limited.

A major challenge in EV-based therapies is the lack of effective delivery systems. It has been reported in several studies that, upon systemic administration, EVs can rapidly distribute throughout the body and accumulate in off-target organs such as liver and spleen. Thus, the therapeutic efficacy of EVs is often limited over time. Moreover, in local administration, EVs can be internalized by macrophages, which may further reduce their therapeutic efficiency (Takahashi et al., 2013; Wiklander et al., 2015; Hu et al., 2021; Xu Z. et al., 2025). For these reasons, biomaterial-based delivery systems have attracted growing interest as carriers for EVs. EV-loaded biomaterials can release EVs in a controlled manner in the implanted area, providing a localized release. In addition, biomaterials can store and improve the stability of EVs by enabling a protective microenvironment in regenerative applications (Murali and Holmes, 2021; Chen et al., 2022; Wang et al., 2024; Yang et al., 2024).

EVs were combined with various biomaterials in several applications. Zhang et al. incorporated EVs derived from dental pulp stem cells (DPSCs) into an injectable fibrin gel for dental pulp regeneration, and they confirmed that EVs promoted cell growth and angiogenesis *in vitro* (Zhang et al., 2020). Zhao et al. combined exosomes from human umbilical vein endothelial cells (HUVECs) with gelatin methacryloyl (GelMA) hydrogel for skin regeneration. It was reported that exosomes showed improved re-epithelization, collagen maturity, and angiogenesis *in vivo* (Zhao et al., 2020). Additionally, therapeutic small extracellular vesicles enriched with Vascular Endothelial Growth Factor A (VEGF-A) and Bone Morphogenetic Protein 2 (BMP-2) mRNAs via a cellular nanoelectroporation system were incorporated into an injectable PEGylated poly(glycerol sebacate) acrylate hydrogel, enabling sustained delivery and enhanced angiogenic, osteogenic, and bone regenerative effects in a rat critical-size defect model (Ma et al., 2023b). In addition, a recent study demonstrated that a 3D-printed hydroxyapatite scaffold functionalized with bone marrow mesenchymal stem cell-derived small extracellular vesicles using fusion peptides as linkers exhibited sustained EV release, enhanced biological activity, and improved bone integration in a critical-sized calvarial defect rat model (Ma et al., 2024). These studies demonstrated the importance of biomaterial-based delivery systems capable of preserving EV bioactivity and enabling sustained release over time. Moreover, silk fibroin (SF)-based scaffolds have been explored as promising EV delivery carriers due to their biocompatibility, ability to incorporate EVs, and tunable release properties (Shi et al., 2017; Bari et al., 2023; Fuest et al., 2024).

SF, a natural protein derived from the cocoons of the *Bombyx mori*, is widely used in regenerative medicine applications (Sultan et al., 2018; Madappura and Madduri, 2023). Due to its unique secondary structure (silk I and silk II), the degradation rate of SF can be adjusted according to the desired need. The control over the degradation rate can provide sustained delivery of therapeutic agents throughout the healing process (Kim et al., 2005; Kim et al., 2012; Pignatelli et al., 2018). The amorphous state (silk I) is water-soluble and a mixture of α-helix and random coil, which can be converted into a crystalline state (water-insoluble) with high β-sheets content (silk II) via several treatments such as water vapor annealing and ethanol. Strong hydrogen bonds are formed within β-sheet structures, providing high mechanical strength. Furthermore, biocompatibility and oxygen permeability of SF further support cell viability at the delivery site, making SF a highly promising biomaterial for EV delivery systems (Hu et al., 2011; Qi et al., 2017). Owing to these properties, SF can be processed into various forms such as hydrogels, nonwovens, and films (Kasoju and Bora, 2012). Recent studies further demonstrated the versatility of SF-based biomaterials in regenerative medicine applications (Sarisoy et al., 2023; Casanova-Batlle et al., 2024). These features make SF an attractive candidate for the development of EV delivery systems. While EV delivery systems have mainly relied on hydrogel-based formulations, dehydrated scaffolds may offer advantages in terms of improved stability and the establishment of long-term release depots (Pritchard and Kaplan, 2011).

**FIGURE 1:**
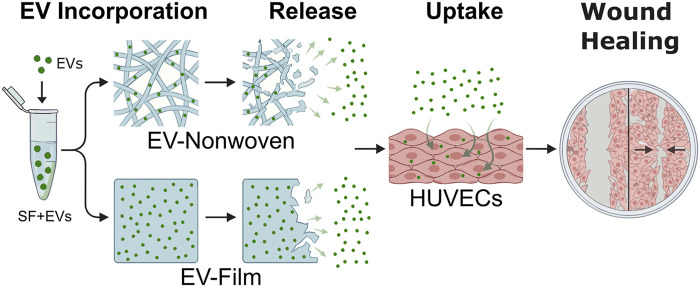
Schematic overview of EV delivery using SF nonwovens and films.

For this purpose, we aimed to develop versatile EV delivery systems based on the natural protein SF capable of providing controlled EV release. To investigate how material architecture and degradation behavior influence EV delivery and biological activity, SF was processed into nonwoven and film structures. While the porous structure of nonwovens mimics the extracellular matrix, films provide a more compact morphology with distinct degradation characteristics (Sell et al., 2007; Lu et al., 2011; Wang et al., 2022). To the best of our knowledge, this is the first study to systematically compare SF nonwovens and films as dehydrated EV delivery systems, enabling a direct assessment of structure-dependent EV delivery behavior in dehydrated SF matrices. To modulate the structural stability and degradation kinetics of SF without compromising EV integrity, a mild water vapor annealing approach was applied. Immortalized gingival fibroblasts (GFs) were selected as a scalable and well-characterized EV source based on their therapeutic potential demonstrated in our previous work (Xu Y. et al., 2025). GF-derived EVs were successfully incorporated into SF nonwovens and films, followed by the investigation of controlled EV release behavior, cellular internalization by HUVECs, and retained bioactivity in a wound healing assay. Overall, this study highlights the potential of dehydrated SF matrices as versatile EV delivery systems for future regenerative medicine applications. A schematic overview of EV delivery using SF nonwovens and films is shown in Figure 1.

## 2 Materials and methods

### 2.1 Fabrication of silk fibroin nonwovens

SF aqueous solution was purchased from the manufacturer (Fibrothelium, Aachen, Germany), which produces the material using its PureSilk technology, ensuring medical-grade quality across a broad range of concentrations. Briefly, SF was separated from sericin by degumming it in a hot alkali solution before dissolving it in a proprietary non-toxic solvent system based on Ajisawa’s reagent. The dissolved fibroin was fully dialyzed against VE water for 8 h by tailored extraction processing. 25% (w/w) SF solutions were kept at −80 °C until further use.

Electrospinning method was performed to produce the nonwovens. Initially, 25% (w/w) SF solution was diluted to 10% (w/w) in ultrapure water (Sartorius, Göttingen, Germany). 5% (w/v) Polyethylene oxide (PEO, 900 kDa, Sigma-Aldrich, St. Louis, MO, USA) solution was prepared in ultrapure water and mixed with 10% SF in weight ratio (SF:PEO, 3:1) to enhance the viscosity of the SF in electrospinning. Once a homogenous solution was obtained, the mixture was electrospun with 12 kV positive voltage and 0.5 ml/h flow rate on a grounded stainless steel mandrel (20 mm diameter) to obtain a uniform and bead-free fiber morphology. The distance between the blunt needle tip (18 G, Vieweg, Kranzberg, Germany) and mandrel was set as 15 cm. During electrospinning process, the mandrel was rotated (360 °/sec) and moved laterally (12 cm) with step and rotational motors (igus, Cologne, Germany) to achieve a homogenous fiber distribution over the mandrel. The electrospinning was performed for 3 h to have 30 ± 10 µm thickness under controlled relative humidity (RH; 30%) and temperature (30 °C) in a chamber (Binder, Tuttlingen, Germany). After the electrospinning process, SF nonwoven was removed by a thin spatula and stored in sealed containers at 4 °C until further use.

### 2.2 Fabrication of silk fibroin films

10% (w/w) SF was obtained by diluting 25% (w/w) SF in ultrapure water. Related amount of SF solution was dragged on a flat Teflon sheet (High-tech-flon, Konstanz, Germany) via a film applicator (Nanotech Industrie Produkte, Berlin, Germany) and dried overnight at room temperature. The gap height of the film applicator was adjusted to 300 µm to have 30 ± 10 µm thickness films after drying. SF film was removed by a scalpel and stored in sealed containers at 4 °C until further use.

### 2.3 Water vapor annealing

To tune the degradation kinetics of nonwovens and films, β-sheet formation was modulated via water vapor annealing. The samples were incubated in a vacuum oven (Hanau, Germany) with RH between 85%-99% at 37 °C for short periods of time (2, 5, and 10 min).

### 2.4 Characterization of nonwovens and films

The morphology of nonwovens and films was characterized by SEM. Initially, samples were sputter coated (Sputter Coater EM SCD500; Leica, Wetzlar, Germany) with a 10 nm gold/palladium layer. Samples were analyzed using a scanning electron microscope Quattro S (Thermo Fisher Scientific, Waltham, MA, USA) with a 10 kV acceleration voltage in a high vacuum environment.

Structural changes in nonwovens and films were characterized by FTIR. Samples were measured in attenuated total reflectance (ATR) mode using GladiATR diamond frontier (PIKE Technologies, WI, USA) equipped with PerkinElmer Spectrum 3 (Waltham, Massachusetts, USA). All measurements were performed in a spectral range from 4000 to 400 cm‐1 with a spectral resolution of 4 cm‐1, accumulating 64 scans at room temperature. The data analysis was carried out with the software Spectrum (Version 10.7.1, PerkinElmer).

### 2.5 Degradation assay

Degradation of nonwovens and films was evaluated for 21 days according to modified ISO 10993-13 standard (International Organization for Standardization, 2010). Initially, films and nonwovens were annealed for 2, 5, and 10 min. After that, they were sterilized with UV treatment each side for 1 h and incubated at 37 °C for 21 days. Degradation media was changed once per week with a fresh complete cell culture medium including Dulbecco’s modified Eagle medium, (DMEM; Thermo Fisher Scientific) substituted with 10% fetal bovine serum (FBS; Gibco, Thermo Fisher Scientific) and 1% antibiotic/antimycotic solution (PAN-Biotech, Aidenbach, Germany). For each time point, the scaffolds were collected and weighed. Accordingly, they were decanted over a pre-weighed filter (Falcon 40 µm cell stainer, Thermo Fisher Scientific) and rinsed 3 times with ultrapure water. After that the scaffolds were dried at 37 °C overnight, and the remaining weight was determined. The degradation was calculated as percentage based on the final weight of the scaffold in comparison to initial scaffold weight.

### 2.6 Cell culture and isolation of gingival fibroblast-derived extracellular vesicles

Immortalized GFs (P10866, Innoprot, Derio (Bizkaia), Spain) were serially cultured at 37 °C, 95% RH, and 5% CO_2_ in conditioned media (CM) composed of media DMEM substituted with 10% exosome-depleted FBS and 1% antibiotic/antimycotic solution. Exosome-depleted FBS was prepared by ultracentrifugation at 110,000 x g for 18 h. EVs were harvested from CM by differential centrifugation according to a modified protocol (Ludwig et al., 2018). Briefly, dead cells, cell debris, and large vesicles were eliminated by centrifugation (5810R, Eppendorf, Hamburg, Germany) at 300 x g for 10 min, 2000 x g for 20 min at 4 °C, and following filtration with 0.20 µm (Corning, NY, USA). The supernatant was mixed with sterile filtered 50% (w/v) polyethylene glycol – 0.375 M sodium chloride (PEG-NaCl) solution for 4:1 volume ratio (CM:PEG-NaCl) to obtain 10% PEG – 0.075 M NaCl final concentration, and incubated overnight at 4 °C. Then, EVs were precipitated at 1,500 x g for 30 min at 4 °C. To remove PEG residues, pellets were washed with sterile filtered phosphate-buffered saline (PBS; Thermo Fisher Scientific), and ultracentrifuged in an Optima LE-80 K ultracentrifuge equipped with SW 32 Ti rotor (Beckman Coulter, Chaska, MN, USA) at 110,000 x g for 70 min at 4 °C. Subsequently, the resulting pellet was washed again with sterile filtered PBS and ultracentrifuged under the same conditions to further eliminate residual PEG (Ludwig et al., 2018). At the end, EVs were resuspended with sterile filtered PBS and stored at - 80 °C in protein LoBind tubes (Eppendorf) to minimize EV adsorption on the tube walls for further use.

### 2.7 Characterization of gingival fibroblast-derived extracellular vesicles

A detailed characterization of EVs derived from immortalized GFs was demonstrated in the previous publication (Xu Y. et al., 2025). Briefly, the size and concentration of EVs were analyzed by nanoparticle tracking analysis (NTA) using a NanoSight NS300 (Malvern Instruments, Worcestershire, UK). EVs were diluted with sterile filtered PBS to ensure that the concentration of detectable particles was within the optimal range of 10^7^–10^9^ particles/mL. For data acquisition, detection threshold and camera level were set as 5 and 12, respectively. Each sample was recorded five times for 60 s to determine the average size and concentration of EVs.

The presence of GF-derived EVs was confirmed by transmission electron microscopy (TEM). EVs were allowed to adsorb on glow discharged formvar-carbon-coated nickel grids (200 mesh, Plano, Wetzlar, Germany) for 5 min. Negative staining was performed after washing in distilled water with 0.5% uranyl acetate (Science Services GmbH, Munich, Germany). Grids were air-dried and imaged using a Hitachi HT7800 transmission electron microscope (Hitachi, Tokyo, Japan) operating at an acceleration voltage of 100 kV.

EV surface markers were detected by Imaging Flow Cytometry (IFCM) as previously described (Ricklefs et al., 2019). Briefly, GF-derived EVs were stained in filtered PBS containing 8% exosome-depleted FBS supplemented with protease-inhibitor and phosphatase-inhibitor. Antibodies used to stain EVs were PE-conjugated anti-CD9 (Biolegend, clone HI9a, 20 μg/mL, diluted 1:30), PacBlue-conjugated anti-CD63 (Biolegend, clone H5C6, 40 μg/ml), FITC-conjugated anti-CD81 (Biolegend, clone 5A6, 40 μg/ml) and an isotype control (MOPC-21, 500 μg/ml). Staining was performed by incubating EVs with antibodies for 45 min at room temperature in the dark. Samples were then washed with 2% exosome-depleted FBS, resuspended, and analyzed using the AMNIS ImageStreamx Mark II Flow Cytometer (AMNIS/Millipore, Seattle). Data analysis was conducted using IDEAS software version 6.2.

### 2.8 GFP-transfection of gingival fibroblasts

Lentivirus encoding plam-GFP was produced in HEK293FT cells by transfection with the packaging plasmids pLP1, pLP2, and pLP/VSVG (Thermo Fisher Scientific, #43-0315). Viral particles were concentrated using a virus precipitation solution (PEG-it, SBI #LV810A-1). 10 µl of concentrated virus were then used to transduce 200,000 GFs cultured in 2 mL of DMEM supplemented with 10% FBS and previously plated in a 6-well plate. One week after transduction, GFP-positive cells were sorted using a BD AriaFusion flow cytometer (BD Biosciences, Franklin Lakes, NJ, USA).

### 2.9 Incorporation of extracellular vesicles

The isolated EVs in filtered PBS initially were pelleted by ultracentrifugation and resuspended in ultrapure water to avoid potential effects of PBS on the production process. The resuspended EVs were vortexed and gently mixed with SF in aqueous phase to produce EV-Nonwoven and EV-Film. A concentration of 1.0 x 10^10^ EVs/ml was used for incorporation. To avoid further dilution, some portion of the ultrapure water was substituted with EVs. Upon mixing, EV-SF mixture was either electrospun or dragged via a film applicator to produce EV-Nonwoven and EV-Film, respectively. Samples were annealed for 10 min to provide water-stability before imaging.

To image EVs incorporated into the nonwoven, electrospinning was performed directly on microscope slides covered with aluminum foil which is punched with 10 mm holes. After that, the fibers were mounted with DAKO fluorescence mounting medium (Agilent Technologies, Santa Clara, CA, USA). EVs incorporated into nonwoven were imaged under confocal microscope (Zeiss LSM 980 Airyscan 2) equipped with a Plan-Apochromat ×63 objective. For this, EVs tagged with green fluorescent protein (EV-GFP) were used which were excited at 488 nm and emission was collected between 500 – 550 nm. Brightfield images of the same samples were acquired using the same objective to image fibers. The software Zen black 2012 (Zeiss, Jena, Germany) was used for image acquisition.

To observe EVs incorporated into film, the samples were initially osmificated in an osmium tetroxide atmosphere. Then, they were embedded in Epon (Serva, Heidelberg, Germany). Polymerization was performed at 90 °C for 2 h and ultrathin sections (70 – 100 nm) were picked up on Cu/Rh grids (HR23 Maxtaform, Plano, Wetzlar, Germany). Contrast was enhanced by staining with 0.5% uranyl acetate and 1% lead citrate (both EMS, Munich, Germany). Samples were examined using a TEM LEO 906 (Carl Zeiss, Oberkochen, Germany), operating at an acceleration voltage of 60 kV.

### 2.10 Extracellular vesicle release assay

A concentration of 1.0 × 10^11^ EVs/ml was incorporated into nonwoven and film, then the scaffolds were annealed for 2 and 10 min respectively to provide water-stability. After that, the samples were cut into proportionally weighed pieces and sterilized by UV irradiation for 1 h on each side. The samples were incubated at room temperature for 14 days on a roller mixer in protein LoBind tubes, using 500 µl of a 1 U/ml protease XIV solution (P5147, 5.5 U/mg, Sigma-Aldrich) prepared in PBS (pH 7.0–7.3). The protease concentration of 1 U/ml was selected to mimic *in vivo* degradation rates (Zhou et al., 2010). The total volume was substituted with fresh protease XIV solution every day. Initially, the samples were vortexed for 5 s to separate EVs, sterile filtered to avoid degradation residues, and stored at – 80 °C for NTA and TEM analysis. The released EVs were detected via NTA and cumulative EV amount-time graph was formed. Additionally, the presence of the released EVs was visualized under TEM. Total degradation was determined at day 14 by weighing the residual material collected on pre-weighed 40 µm filters, after three rinses with ultrapure water and drying overnight at 37 °C. EV release efficiency was calculated by comparing the experimentally quantified EV amount obtained by NTA with the theoretical EV amount expected from the degraded fraction of the EV-loaded SF materials.

### 2.11 *In vitro* extracellular vesicle uptake assay

HUVECs were isolated from human umbilical cords as previously described (Moreira et al., 2015). Human umbilical cords were obtained after written informed consent at University Hospital Aachen, Germany and were provided by the RWTH Aachen University Centralized Biomaterial Bank (cBMB), in compliance with its regulations, following RWTH Aachen University Medical Faculty Ethics Committee approval (cBMB project number 323). HUVECs were cultured in endothelial growth medium 2 (EGM2, PromoCell, Germany) supplemented with FBS, antibiotic/antimycotic solution, epidermal growth factor, basic fibroblast growth factor, insulin-like growth factor, vascular endothelial growth factor 165, ascorbic acid, heparin, and hydrocortisone. The cells were expanded at 37 °C in a humidified environment with 5% CO_2_, and cell passages up to 5 were used for the experiments. After HUVECs reached 80% – 90% confluency, they were trypsinized and resuspended with EGM2 to obtain the desired cell concentration.

Meanwhile, 1 x 10^11^ EVs/ml of EVs were labelled with PKH26 fluorescent dye according to manufacturer´s instructions (Sigma-Aldrich). Excess dye was removed through washing and ultracentrifugation. After that, EVs labelled with PKH26 dye were incorporated into dissolvable SF nonwoven and film structures to produce EV-Nonwoven and EV-Film as described above. As a control group, PKH26 staining was performed in the same way, but ultrapure water was used instead of EVs. The samples were cut and placed in chamber slides (8 wells, Nunc Lab-Tek, Sigma-Aldrich) and stabilized with punched (0.5 cm^2^) silicone molds on top. HUVECs with the concentration of 2.5 x 10^4^ cells/ml were seeded on EV-Nonwoven and EV-Film and incubated for 12 h at 37 °C (5% CO_2_). After that, the samples were washed with PBS and fixed in 4% paraformaldehyde (Carl Roth, Karlsruhe, Germany) for 1 h at room temperature. The cells were then washed three times with PBS and permeabilized with 0.1% Triton X-100 (Sigma-Aldrich) for 5 min. Actin filaments were stained with phalloidin-iFluor 488 conjugate (Cayman Chemicals, Ann Arbor, MI, USA) in 1% (w/v) bovine serum albumin (Sigma-Aldrich) for 90 min at room temperature. After washing the cells three times with PBS, the nuclei were stained with DAPI (Carl Roth) for 15 min and washed again three times with PBS. Finally, the stained HUVECs were visualized under confocal microscope (Opera Phenix HH2400, PerkinElmer) equipped with a Plan-Apochromat ×40 objective. For the visualization of PKH26 dye, the samples were excited with a 551 nm laser line, and emission was collected at 567 nm. For DAPI staining, the samples were excited at 405 nm laser line, and emission was obtained at 410–495 nm, while for phalloidin-iFluor 488, the excitation was performed at 488 nm, and emission was acquired at 495–630 nm. The software Harmony 5.2 (PerkinElmer) was used for image acquisition and analysis.

### 2.12 Wound healing assay

As HUVECs play a critical role in wound healing, the wound healing assay was performed with HUVECs (Gonzalez et al., 2016). Initially, HUVECs were seeded at a concentration of 3.0 x 10^5^ cells/mL in ibidi culture inserts (2-well, ibidi GmbH, Graefelfing, Germany), which were placed in ibidi µ-Slides (8-well, ibiTreat polymer coverslip, ibidi GmbH) and incubated overnight at 37 °C with 5% CO_2_ to achieve a confluent monolayer. Following the removal of the ibidi culture inserts, the cells were washed with PBS. Subsequently, 250 µl of FBS-free EGM2 supplemented with EV-Nonwoven, EV-Film, or free EVs was added to the cells for each condition. For this, EV-Nonwoven and EV-Film were produced with an EV concentration of 9.1 x 10^11^ EVs/ml corresponding to a theoretical EV amount of 0.9 x 10^10^ EVs upon dissolution. Accordingly, a comparable EV concentration of 1.0 x 10^10^ EVs/ml was used for the free EV group. The materials were dissolved in EGM2, sterile-filtered, and centrifuged at 10,000 × g for 10 min to remove residual material prior to cell incubation. As a control, an equal amount of ultrapure water was used. The process of wound healing was monitored by the JuLI Stage (Real-Time Cell History Recorder, NanoEntek, Seoul, South Korea) for a period of 24 h at 37 °C with 5% CO_2_. The confluence was calculated in wound healing mode for 1-h imaging intervals, with a sensitivity of 9 and a background level of 2.

### 2.13 Statistical analysis

All data are presented as the mean ± standard deviation (SD). Statistical analysis was performed using Student’s t-test or one-way analysis of variance (ANOVA) followed by the Holm–Šidák *post hoc* test. A *p*-value < 0.05 was considered statistically significant (* *p* < 0.05, ** *p* < 0.01, *** *p* < 0.001).

## 3 Results

### 3.1 Fabrication and characterization of silk fibroin nonwovens and films

**FIGURE 2:**
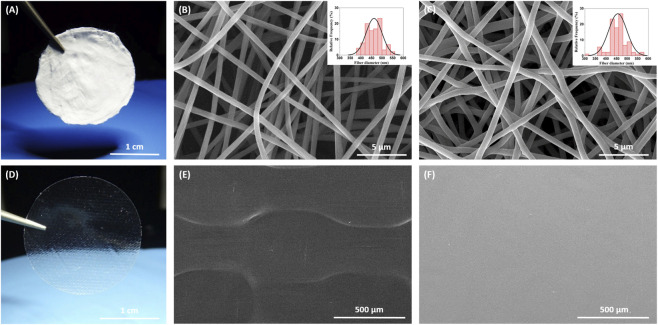
Morphological characterization of SF nonwovens and films. **(A)** Macroscopic image of SF nonwoven. **(B)** Fiber morphology and distribution under SEM. **(C)** Fiber morphology and distribution after 10 min of water vapor annealing. **(D)** Macroscopic image of SF film. **(E)** Surface morphology under SEM. **(F)** Surface morphology after 10 min of water vapor annealing.

Nonwovens were fabricated via electrospinning method as shown macroscopically in Figure 2A. SEM analysis showed a homogeneous fiber morphology with uniformly distributed, smooth, and bead-free fibers. The average fiber diameter was measured as 462.3 ± 37.5 nm (Figure 2B). To tune the degradation rate of SF, mild water vapor annealing (37 °C) was applied at different time points (2, 5, and 10 min). After the annealing, no significant change in the average fiber diameter was observed, with the dimensions of 452.4 ± 47.4 nm, 468.9 ± 42.1 nm, and 455.8 ± 42.7 nm, respectively (Supplementary Figure 1). In addition, fibers flattened after 10 min of treatment by preserving their homogenous fiber morphology and uniform distribution (Figure 2C). Films were produced homogenously by casting silk fibroin solution on a Teflon sheet, which provided grooved structures (Figures 2D, E). When films were exposed to water vapor annealing, the surface became smooth and flat (Figure 2F).

**FIGURE 3:**
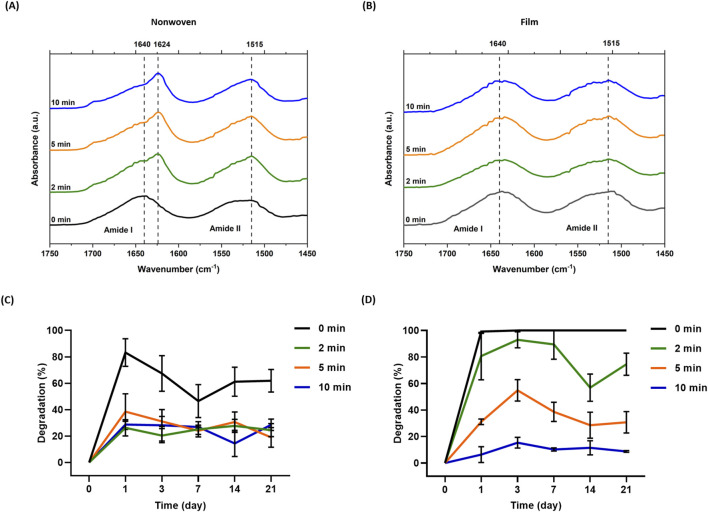
Structural changes and degradation behavior of SF nonwovens and films. **(A, B)** FTIR spectra of SF nonwovens **(A)** and SF films **(B)** following water vapor annealing for 0, 2, 5, and 10 min. **(C, D)** Degradation behavior of SF nonwovens **(C)** and SF films **(D)** treated with different water vapor annealing durations and incubated in complete cell culture medium over 21 days.

Structural changes of SF nonwovens and films at different annealing durations were characterized by FTIR (Figures 3A, B). The amide I (1600 – 1700 cm^-1^) and amide II (1450 – 1600 cm^-1^) regions were chosen to observe the formation of β-sheets. Initially, the peak was centered at 1640 cm^-1^ for untreated nonwoven, which stands for α-helix and random coil structures. With increasing annealing time, the peak gradually shifted to 1624 cm^-1^, indicating a conformational change to β-sheets. Similarly, a peak at 1515 cm^-1^ gradually appeared with increasing annealing time, reflecting the transformation of random coils to β-sheets (Min et al., 2006; Hu et al., 2011). However, although morphological changes were observed under SEM following annealing, no significant shift in the FTIR peaks was detected at any treatment point under mild annealing conditions (37 °C, 2–10 min) compared with the untreated film.

### 3.2 Degradation assay

To analyze the effect of water vapor annealing on the degradation kinetics of SF nonwovens and films, the materials were incubated in complete cell culture medium supplemented with FBS and antibiotic/antimycotic solution for 21 days. The degradation rate decreased following annealing for both groups. The untreated nonwoven degraded 61.9% ± 8.5 % after 21 days, whereas the degradation rate decreased upon annealing (Figure 3C). No notable difference was observed among the different annealing time points of SF nonwoven. On the other hand, although the untreated film degraded completely, the degradation rate was reduced to 74.5% ± 8.3 % and 30.8% ± 8.1 % with 2- and 5-min treatments after 21 days, respectively (Figure 3D). In addition, the 10-min-treated film was mostly stable in the complete cell culture medium, and only lost 8.7% ± 0.6 % of its original weight over time. Notably, SF films showed surface smoothing under SEM and reduced degradation following annealing while no significant FTIR peak shift was detected. Lastly, for both treated nonwovens and films, the majority of the degradation occurred within the first day and remained relatively constant throughout the degradation period.

### 3.3 Characterization of gingival fibroblast-derived extracellular vesicles

**FIGURE 4:**
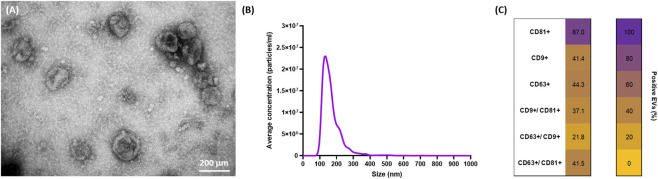
Characterization of GF-derived EVs. **(A)** Morphology of EVs derived from GFs under TEM. **(B)** The average concentration-size distribution of EVs derived from GFs under NTA. **(C)** IFCM analysis of the EV-associated surface markers CD81, CD63, and CD9.

EVs derived from GFs were characterized under TEM and NTA. TEM images revealed a characteristic cup-shaped morphology of EVs (Figure 4A). The size distribution was determined by NTA, which mainly ranges between 100 nm and 300 nm with a mean diameter of 167.8 ± 1.2 nm (Figure 4B). IFCM confirmed the presence of the key EV-associated surface markers CD81, CD63, and CD9 (Figure 4C). Among these, CD81 was identified as the most predominant tetraspanin (87.0% ± 12.8 %) in GF-derived EVs.

### 3.4 Incorporation of extracellular vesicles

**FIGURE 5:**
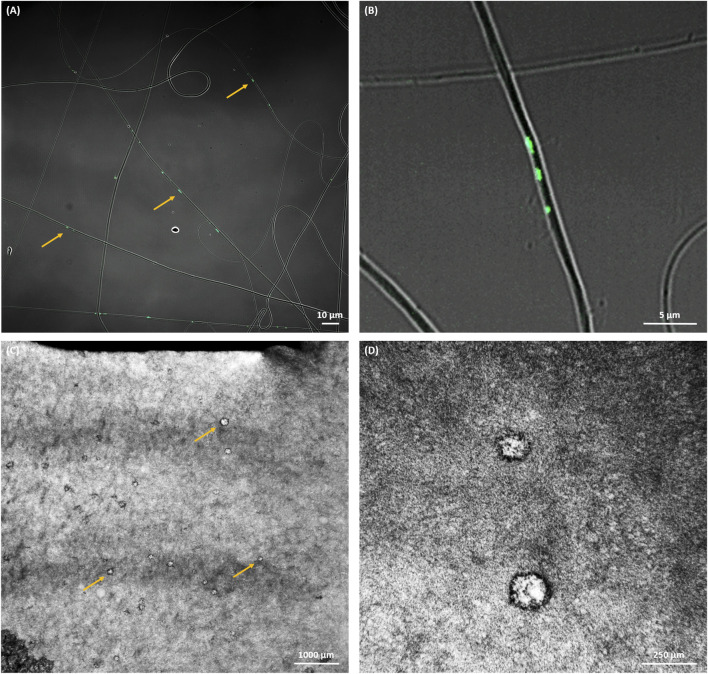
EV incorporation into SF nonwovens and films. **(A)** GFP-labelled EVs incorporated into water vapor annealed EV-Nonwoven observed by confocal microscopy. **(B)** Close-up image of the EVs within the single fibers of EV-Nonwoven. **(C)** EVs incorporated into annealed EV-Film under TEM. **(D)** Close-up image of the EVs within EV-Film.

EVs at a concentration of 1.0 × 10^10^ EVs/ml were mixed with the silk fibroin solution, electrospun, and subsequently annealed for 10 min to provide water-stability. The incorporation of EVs did not influence the electrospinning process and homogenous fibers were obtained (Figure 5A). Due to GFP-labelling, they could be imaged under confocal microscope within the fibers. Since the average diameter of the EVs (167.8 ± 1.2 nm) was smaller than the average fiber diameter (455.8 ± 42.7 nm), EVs were successfully encapsulated within the single fibers during the electrospinning process. Additionally, EVs preserved their structure and were aligned within the fibers. A group of EVs are demonstrated within a single fiber in Figure 5B. Similarly, EVs with the concentration of 1.0 x 10^10^ EVs/ml was mixed gently with SF in the aqueous phase, cast and annealed for 10 min. To investigate EV incorporation into film, samples were observed under TEM (Figure 5C). EVs were successfully incorporated into the film by keeping their lipid bi-layer intact (Figure 5D). In addition, EVs were evenly distributed within the film.

### 3.5 Release of extracellular vesicles

**FIGURE 6:**
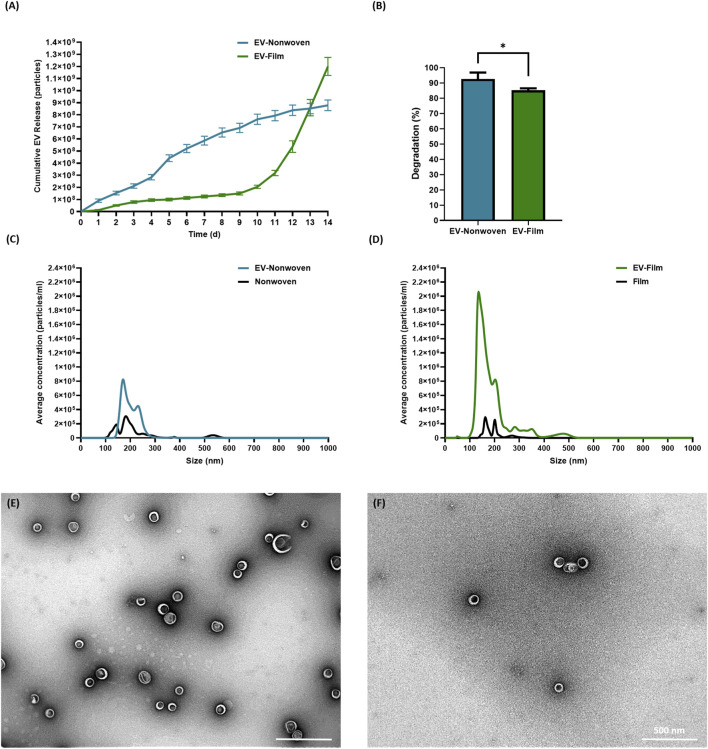
Release of EVs from water vapor annealed SF nonwovens and films. **(A)** Cumulative EV release from EV-Nonwoven and EV-Film in Protease XIV solution over 14 days. **(B)** Degradation of EV-Nonwoven and EV-Film after 14 days (*p < 0.05). **(C, D)** The comparison of average concentration-size distribution of EV-Nonwoven at day 6 **(C)** and EV-Film **(D)** at day 12 with control groups. **(E, F)** Visualization of the EVs released from EV-Nonwoven **(E)** and EV-Film **(F)** under TEM.

The cumulative release kinetics of EVs from water vapor annealed EV-Film and EV-Nonwoven were investigated over 14 days (Figure 6A). EVs were released in different kinetics from nonwovens than films. EV-Nonwoven showed a more controlled release than EV-Film over 14 days. The released EVs reached 5.9 x 10^8^ ± 3.7 x 10^7^ EVs at day 7 and 8.8 x 10^8^ ± 4.4 x 10^7^ EVs after 14 days. In contrast, EV-Film initially released EVs at a comparatively slower rate until day 9, which is 1.5 x 10^8^ ± 1.3 x 10^7^ EVs, however releasing 1.2 x 10^9^ ± 7.4 x 10^7^ EVs at day 14. Additionally, significantly more degradation occurred in nonwovens (92.7% ± 4.2 %) than films (85.2% ± 1.3 %) after 14 days (Figure 6B; *p* = 0.042). The release efficiency of EV-loaded SF materials was calculated relative to the initially incorporated EV amount. Based on the initial EV concentration of 1.0 × 10^11^ EVs/ml in EV-Nonwoven and EV-Film, theoretical EV release amounts of 1.7 × 10^9^ ± 7.9 x 10^7^ EVs and 1.6 × 10^9^ ± 2.4 x 10^7^ EVs were expected based on the weight of cut samples and the degradation rates of 92.7% ± 4.2 % for EV-Nonwoven and 85.2% ± 1.3 % for EV-Film, respectively. Comparing the experimentally detected EV amounts with these theoretical values resulted in release efficiencies of 50.9% ± 3.4 % for EV-Nonwoven and 75.5% ± 4.8 % for EV-Film. A quantitative comparison of degradation behavior and EV release efficiency is summarized in Table 1.

**TABLE 1:** Quantitative comparison of degradation behavior, EV release efficiency, and wound healing performance between EV-Nonwoven and EV-Film.

Group	Degradation (%)	EV release efficiency (%)	Wound healing (%)
EV-Nonwoven	92.7 ± 4.2	50.9 ± 3.4	41.5 ± 3.6
EV-Film	85.2 ± 1.3	75.5 ± 4.8	28.0 ± 1.1

To understand whether the detected particles under NTA were EVs, the average concentration-size distribution was analyzed on specific days and compared to the control groups without EVs in Figures 6C, D. Both the degradation solution from EV-Nonwoven and EV-Film showed clearly higher particle concentration than the control groups in the range of 100 – 300 nm on day 6 and 12, respectively, demonstrating that the detected particles were EVs. The EV-size distribution was mainly preserved for both groups after the release. Moreover, the released EVs were successfully visualized under TEM in Figures 6E, F.

### 3.6 Uptake of extracellular vesicles

**FIGURE 7:**
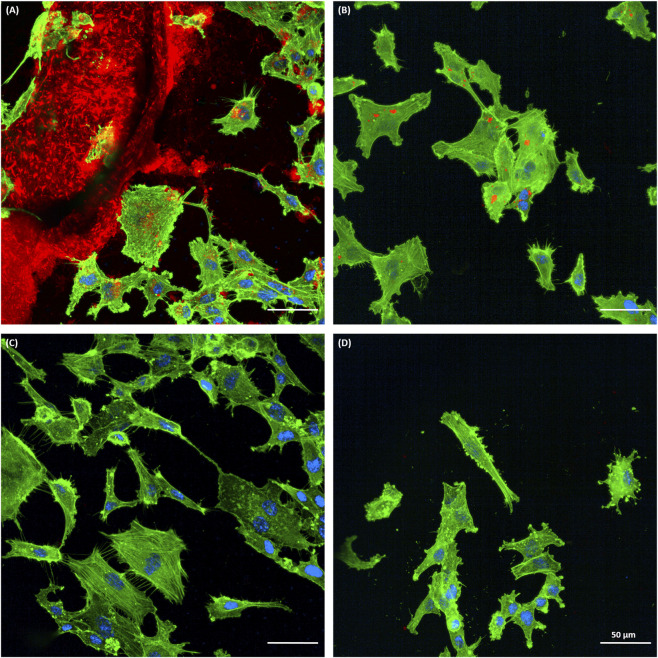
Uptake of PKH26-labelled EVs by HUVECs. **(A)** Internalization of EVs released from EV-Nonwoven by HUVECs visualized under confocal microscopy. **(B)** Internalization of EVs released from EV-Film by HUVECs. **(C, D)** HUVECs cultured with nonwoven **(C)** and film **(D)** materials without EVs as control groups, respectively. The actin cytoskeleton and nuclei were stained with phalloidin-488 (green) and DAPI (blue), respectively. EVs were labelled with PKH26 fluorescent dye (red).

After demonstrating that the incorporated EVs were successfully released from EV-loaded nonwovens and films by preserving their lipid bi-layer intact, the uptake of the released EVs was investigated in Figure 7. For this, EVs with the concentration of 1.0 x 10^11^ EVs/ml were initially stained with PKH26 dye and incorporated into nonwovens and films. After that HUVECs were seeded directly on top of the samples and incubated for 12 h. EV-Nonwoven dissolved mostly and a remnant of EV-Nonwoven can be seen in Figure 7A. HUVECs showed a well-spread morphology and nicely attached to EV-Nonwoven. Upon dissolution, EVs were released from the structure and were successfully internalized by HUVECs, which localized toward their nuclei. Similarly, upon dissolution of EV-Film, EVs were internalized by HUVECs and aggregated nearby nuclei (Figure 7B). The well-spread morphology was observed as well with HUVECs incubated with EV-Film for 12 h. To distinguish EV uptake from potential PKH26 dye aggregation artifacts, the experiment was additionally performed using PKH26 dye without EVs as a control group. As shown in Figures 7C, D, no nonspecific particle uptake by HUVECs and no detectable fluorescence signal within nonwovens or films were observed under identical imaging conditions. These findings suggest that PKH26-labelled EVs were successfully released from EV-Nonwoven and EV-Film and subsequently internalized by HUVECs.

### 3.7 Wound healing

**FIGURE 8:**
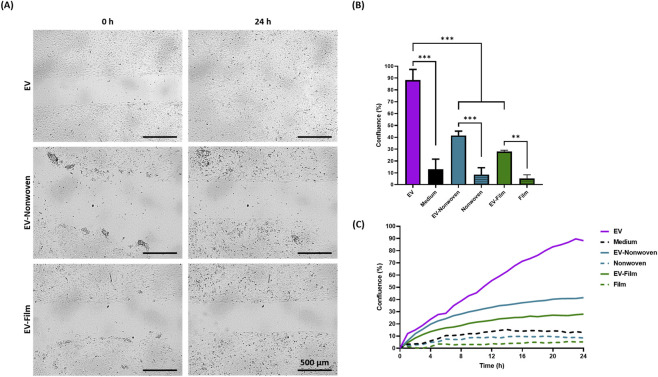
**(A)** Wound healing assay with HUVECs incubated with EV, EV-Nonwoven, and EV-Film at 0 and 24 h. **(B)** Quantitative analysis of confluence (%) at 24 h (*p < 0.05, **p < 0.01, ***p < 0.001). **(C)** Quantitative analysis of confluence (%) over 24 h by JuLI Stage (Real-Time Cell History Recorder).

To investigate the retained functionality of EVs after release from material, a wound healing assay was performed on HUVECs under FBS-free conditions in Figure 8. When free EVs were added in the medium at a comparable concentration of 1.0 x 10^10^ EVs/ml, the migration of HUVECs was significantly enhanced, reaching 88.2% ± 9.0 % (*p* < 0.001) confluence in 24 h (Figures 8A, B). Similarly, a substantial enhancement was achieved when the cells were incubated with EV-Nonwoven and EV-Film groups, reaching 41.5% ± 3.6 % (*p* < 0.001) and 28.0% ± 1.1 % (*p* = 0.004) confluence, respectively. On the other hand, when HUVECs were incubated in an FBS-free environment without any EVs, the cells reached 12.9% ± 8.6 %, 8.5% ± 5.8 %, and 5.3% ± 3.1 % confluence within 24 h in the medium, nonwoven and film groups, respectively (Figure 8C, Supplementary Figure 2). Therefore, EVs released from the delivery system significantly improved the migration of HUVECs in the wound healing assay compared to the control. A quantitative comparison of wound healing performance, degradation behavior, and EV release efficiency is summarized in Table 1.

## 4 Discussion

The therapeutic potential of EVs has been well recognized due to their favorable advantages over cell-based therapies, however, their rapid clearance and phagocytic uptake by macrophages following systemic or local administration remain as major challenges for clinical applications (Takahashi et al., 2013; Wiklander et al., 2015; Hu et al., 2021; Xu Z. et al., 2025). To address these limitations, EV delivery systems have been proposed to provide localized and sustained release of EVs (Murali and Holmes, 2021; Chen et al., 2022). Recent studies on protein-based biomaterials and engineered therapeutic systems support the development of multifunctional biomaterial-assisted therapeutic platforms with tunable biological interactions and enhanced application versatility (Guo et al., 2025; Wen et al., 2025; Wang et al., 2026). In this study, SF was selected as a biomaterial carrier for an effective EV delivery system due to its tunable degradation kinetics, oxygen permeability, and biocompatibility, which allow precise modulation of EV release while supporting cell viability at the delivery site (Pritchard and Kaplan, 2011; Qi et al., 2017). SF was produced in the form of nonwovens and films to investigate how material structure and degradation behavior affect EV delivery and biological activity in dehydrated SF matrices. To precisely modulate the degradation kinetics of SF without compromising EV integrity, a mild water vapor annealing technique was selected. This approach has been reported as compatible with sensitive biomolecules, such as growth factors, and suitable for enabling controlled release (Dinis et al., 2014; Pignatelli et al., 2018). Accordingly, SF nonwovens and films were treated by water vapor annealing, and the degradation rate was tuned by varying the annealing duration between 2 and 10 min at 37 °C.

Water vapor annealing induced similar morphological changes in SF nonwovens and films. After 10 min of treatment, no significant change in the fiber diameter was observed for the nonwovens, however, surface smoothing and flattening were evident in both groups, which is consistent with previously reported post-treatment effects of SF (Meinel et al., 2009; Reizabal et al., 2022) (Figure 2).

Since the secondary structure of SF is a key parameter to tune the release kinetics of EVs, conformational changes were further analyzed at different treatment durations (2, 5, and 10 min). While comparable morphological responses were observed for both groups under SEM, distinct structural changes were obtained in SF nonwovens and films in FTIR, highlighting the influence of material architecture on structural rearrangement. In SF nonwoven, progressive peak shifts at 1624 cm^-1^ and 1515 cm^-1^ were observed with increasing annealing time, indicating an increase of β-sheets within the structure (Figure 3A) (Kim et al., 2003; Alessandrino et al., 2008). In contrast, comparable changes were not clearly detected for SF film under the same annealing conditions (Figure 3B), despite notable morphological smoothening and slower degradation rates being observed (Figure 2F and Figure 3D). This could be attributed to the dense and compact structure of SF films, which may limit vapor diffusion and reduce molecular mobility compared with the highly porous nanofibrous network of SF nonwoven. Under mild processing conditions such as low temperature (37 °C) and short annealing durations (2 – 10 min), SF film might undergo partial conformational rearrangement toward a more stabilized structure without forming a fully β-sheet-dominated Silk II detectable by FTIR. Previous reports have described similar behavior, where water vapor annealing with low temperature and short durations promoted an insoluble helix-dominated silk I structure prior to the transition of β-sheet-dominated silk II structure, which needs higher temperature and longer annealing time to provide sufficient energy for silk I to silk II transition (Lu et al., 2010; Hu et al., 2011). Therefore, annealed SF film demonstrated intermediate structural stabilization under mild annealing conditions, which correlated with morphological smoothening and decreased degradation rate despite limited changes in β-sheet-related FTIR peaks.

Importantly, the observed slower degradation rates following annealing provides functional evidence of enhanced structural stability for both SF nonwoven and film (Figures 3C, D). Although annealing considerably reduced the degradation of both groups, SF nonwoven was more prone to β-sheet formation due to its highly porous morphology, which correlates with FTIR peak shifts (Al-Enizi et al., 2018). Since most of the weight loss occurred at day 1 and stayed in a constant range for both groups over 21 days, it can be concluded that the degradation of SF was mainly driven by the dissolution of SF protein in the medium (Hines and Kaplan, 2011).

Consistent with the recommendations of the International Society for Extracellular Vesicles (ISEV), GF-derived EVs were small (< 200 nm), exhibited their characteristic cup-shaped morphology, and expressed the key EV-associated surface markers CD81, CD63, and CD9 (Figure 4) (Thery et al., 2018). Notably, CD81 was identified as the predominant tetraspanin in GF-derived EVs, suggesting its potential role in EV–cell interactions and intercellular communication (Zhang and Grizzle, 2014). The presence of characteristic EV-associated surface markers further supports the suitability of GF-derived EVs for incorporation into biomaterial-based delivery systems.

In recent years, EVs have been incorporated into various hydrogels to enable controlled release and sustained therapeutic effects. In these studies, EVs were either added to the system after the production of the biomaterial or directly encapsulated during the production process (Shi et al., 2017; Zhang et al., 2020; Zhao et al., 2020; Bari et al., 2023). In addition, EVs were often stabilized in hydrated environments due to hydrogel-based systems which can provide a stable osmotic environment. However, it has been demonstrated that EVs can withstand dehydrated conditions as well, such as lyophilization, when sufficient osmotic stabilization is provided. Therefore, the dehydrated state could preserve the structure of EVs for longer periods of time and extend the shelf life without compromising their bioactivity (Yuan et al., 2021; Trenkenschuh et al., 2022; Geng et al., 2025). Moreover, it was recently reported that EVs were electrospun with polyvinylpyrrolidone to preserve the stability of EVs and improve storage (Nemeth et al., 2022).

In our study, we aimed to enhance the bioactivity of SF and achieve a sustained release by encapsulating GF-derived EVs within SF nonwoven and film structures. GF-derived EVs were selected as a well-characterized EV source, as their biomolecular profile and therapeutic potential have been comprehensively investigated in our previous work, demonstrating pronounced regenerative effects (Xu Y. et al., 2025). In addition, the immortalized GF source provided a scalable and robust EV production, offering a controlled starting point for investigating dehydrated SF-based EV delivery systems. Therefore, the presented EV delivery system is not restricted to GF-derived EVs, but can also be readily extended to other EV sources for different clinical applications.

Confocal microscopy revealed that GFP-tagged EVs were successfully incorporated within the individual fibers of SF nonwoven (Figures 5A, B). Notably, EVs maintained their integrity despite exposure to the high electrostatic field applied during the electrospinning (+ 12 kV). Additionally, no common toxic solvents such as HFIP (1,1,1,3,3,3-Hexafluoro-2-propanol) were used during electrospinning process, instead a greener alternative PEO was selected, which further supported EV integrity (Kopp et al., 2020). This observation is particularly relevant, as EVs might be susceptible to physical and chemical stresses (Sivanantham and Jin, 2022). However, under mild process parameters, a SF-based electrospinning can be compatible for an EV delivery system. Moreover, EV incorporation into SF films further confirmed the ability of SF to retain EVs within a dehydrated environment. Following casting, EVs were uniformly distributed throughout the film and preserved their lipid bi-layer structure as it was visualized by TEM (Figures 5C, D). Importantly, morphology of EVs remained unaffected by 10 min of water vapor annealing (85% – 99% RH at 37 °C) for both EV-Nonwoven and EV-Film groups, demonstrating that this mild post-treatment does not compromise EV integrity. Overall, these findings indicate that, with careful consideration of SF processing steps such as electrospinning, casting, and water vapor annealing, a safe EV delivery system can be produced without compromising EV integrity. This structural preservation is a prerequisite for maintaining EV bioactivity and supports the suitability of SF nonwovens and films as biomaterial carriers for EV delivery applications (Leung et al., 2022).

It has been reported that the release of small molecules entrapped in SF nonwovens and films is mainly driven by diffusion (Tomoda et al., 2020; Gough and Hu, 2021). Smaller diffusion coefficient was obtained in crystallized SF than untreated SF, and an inverse correlation was observed between the molecular weight of the released molecule and diffusion coefficient (Hines and Kaplan, 2011). On the other hand, to release larger structures such as EVs, either a sufficient mesh porosity for diffusion or degradation is necessary (Zhang et al., 2020; Zhao et al., 2020; Tang et al., 2022; Bari et al., 2023; Ma et al., 2023a). In Figure 6A, annealed EV-Nonwoven and EV-Film showed different EV release characteristics. While EVs were released in a more sustained way from EV-Nonwoven over time, an initially slower release of EVs followed by a faster release at later time points was observed from EV-Film. It could be explained by the microstructure of the nonwovens. Nonwovens exhibit high porosity and surface-area-to-volume ratio. However, films have more compact surface structure than the nonwovens, therefore, the EVs were released in a more controlled manner from nonwovens (Gough and Hu, 2021). Quantitative analysis demonstrated higher EV release efficiency for EV-Film (75.5% ± 4.8 %) compared to EV-Nonwoven (50.9% ± 3.4 %) in Table 1. This may be associated with increased EV retention within the highly porous structure of EV-Nonwoven. Furthermore, the reported EV release efficiencies likely represent conservative estimates, as additional EV losses may have occurred during sterile filtration for NTA measurements and through possible EV adsorption to residual SF fragments and other surfaces under test conditions. In addition, the released EVs from both EV-Nonwoven and EV-Film maintained their characteristic cup-shaped structure (Figures 6E, F), indicating preservation of EV integrity after incorporation and release (Corona et al., 2023). Consistent with this observation, SF processed in different forms has been reported to stabilize the active ingredients within its structure and preserved their integrity in various drug and gene delivery applications (Tomeh et al., 2019). Together, the two different release characteristics obtained in this study suggest that SF matrices can be tailored to meet different therapeutic requirements, which could potentially benefit diverse clinical applications.

The uptake of the released EVs from EV-Nonwoven and EV-Film was examined using HUVECs to assess whether EV bioavailability and internalization were preserved after incorporation and release. In both groups, EV uptake was successfully observed after 12 h of incubation (Figures 7A, B), which is consistent with previous reports showing maximal internalization of EVs by endothelial cells within this time frame (Zhang et al., 2020; Xu Y. et al., 2025). Confocal images revealed that EVs released from EV-Nonwoven and EV-Film were internalized by HUVECs and accumulated in the perinuclear region, a characteristic localization associated with endocytic uptake pathways (Mulcahy et al., 2014; Chae et al., 2024). In addition, the perinuclear accumulation indicates an active internalization rather than passive surface association, which supports the preservation of bioavailability after release. Notably, both EV-Nonwoven and EV-Film supported a well-spread morphology and strong interactions of HUVECs, therefore providing a favorable cellular microenvironment. This finding supports previous research that has reported improved endothelial responses upon EV uptake (Clos-Sansalvador et al., 2022). To minimize false-positive signals associated with PKH26 dye aggregation or nonspecific dye transfer, the same experimental procedure was performed using PKH26 dye without EVs as a control group, and no detectable fluorescence signal was observed under identical imaging conditions (Figures 7C, D) (Takov et al., 2017; Puzar Dominkus et al., 2018). Thus, the observed fluorescence predominantly originated from released PKH26-labelled EVs internalized by HUVECs.

It has been demonstrated in numerous studies that EVs derived from different cell sources are internalized by recipient cells and promote wound healing via several molecular mechanisms (Hade et al., 2022). For instance, Glady et al. showed that human keratinocyte-derived EVs enhanced cell proliferation and migration by activating the MAPKinase pathway (Glady et al., 2021). In our previous work, the molecular content and functional effects of EVs derived from different sources, including GFs and dental pulp stem cells were characterized in detail. Both EVs derived from GFs and dental pulp stem cells exhibited comparable performance in functional assays (Xu Y. et al., 2025). In addition, recent studies demonstrated that engineering EV production methods, including hypoxic preconditioning and 3D culture systems, can enhance the regenerative potential of EVs through improved wound healing (Deng et al., 2024). Moreover, advanced delivery approaches designed to enhance therapeutic efficacy and localized delivery have become increasingly important for endothelial regeneration and regenerative medicine (Shen et al., 2025; Wang et al., 2025; Zheng et al., 2025). Thus, localized and sustained EV delivery using SF-based biomaterials may represent a promising strategy for wound healing applications.

HUVECs play a crucial role in wound healing, particularly through angiogenesis, which supplies oxygen and nutrients to newly formed tissue, as well as through endothelial cell migration to promote wound closure. They are involved in all phases of wound healing from inflammation to tissue remodeling (Yi et al., 2020; Morbidelli et al., 2021). Therefore, a wound healing assay was conducted using HUVECs to analyze the function of EVs derived from GFs after incorporation and release from EV-Nonwoven and EV-Film. The wound healing was significantly improved after 24 h when HUVECs were incubated with free EVs, confirming their pro-regenerative effect (Figure 8) (Bruno et al., 2019a; b). Similarly, incubation with EV-Nonwoven and EV-Film notably enhanced wound closure, indicating that EVs largely retained their function after the release. Interestingly, although EV-Nonwoven showed lower EV release efficiency (Table 1), it induced greater wound healing activity compared to EV-Film. This suggests that the nanofibrous and highly porous structure of the nonwoven may preserve EV bioactivity more effectively than the more compact film structure. However, despite the use of comparable EV concentrations, the wound healing effect observed in HUVECs was relatively greater in the free EV group compared with the EV-Nonwoven and EV-Film groups. This could be explained by the EV incorporation process, where both high voltage applied during electrospinning for nonwovens and extended drying for films may have partially affected the EV stability and functionality. In particular, exposure to electrical fields during electrospinning and extended dehydration during film preparation may induce structural stress on EVs during processing. Nevertheless, the enhanced wound healing observed in both EV-Nonwoven and EV-Film groups indicates that substantial EV bioactivity was preserved after incorporation and release.

Based on the preserved functionality observed in this study, dehydrated SF matrices may also provide advantages regarding storage and long-term stability. Due to the dehydrated state of EV-loaded nonwovens and films, such systems may reduce the dependence on ultra-low temperature storage conditions such as at −80 °C and support improved handling and transportation of EV formulations (Yuan et al., 2021; Geng et al., 2025). In addition, the scalable processing of SF into nonwoven and film structures, together with the compatibility of these systems with sterilization procedures, may support the reproducible fabrication of EV-loaded biomaterials for larger-scale applications. In the present study, EV-loaded SF materials were sterilized by UV exposure and stored at 4 °C prior to the experiments while maintaining EV bioactivity, as demonstrated by EV internalization by HUVECs and the retained functional effect in the wound healing assay. This suggests that dehydrated SF matrices may contribute to improved shelf-life and storage stability of EV-based therapeutics. Future studies focusing on long-term storage and preservation of EV bioactivity under clinically relevant conditions would further improve the understanding and future development of dehydrated SF matrices in EV delivery systems.

## 5 Conclusion

In this study, SF was investigated as a versatile biomaterial carrier for the controlled delivery of EVs. By processing SF into nonwoven and film structures and modulating their degradation kinetics through a mild water vapor annealing approach, distinct structure-dependent EV release behaviors were achieved. EVs were successfully incorporated into both SF nonwovens and films, released with different kinetics over time, and internalized by HUVECs while maintaining their structural integrity and bioactivity. Furthermore, EV-Nonwoven and EV-Film significantly enhanced HUVEC migration in an *in vitro* wound healing assay, indicating preserved EV functionality after release from the delivery system. Overall, these findings highlight the potential of dehydrated SF matrices as promising systems for controlled EV delivery and support their further development for future regenerative medicine applications.

## Data Availability

The original contributions presented in the study are included in the article/Supplementary Material, further inquiries can be directed to the corresponding author.
