# Supplementary material for: Dehydrated silk fibroin matrices as versatile delivery systems for extracellular vesicles
Source: Front Bioeng Biotechnol. 2026 Jul 7;14:1873981. doi: 10.3389/fbioe.2026.1873981 (PMC13385103; doi:10.3389/fbioe.2026.1873981)
Supplement: Supplementary file 1 [file DataSheet1.docx]

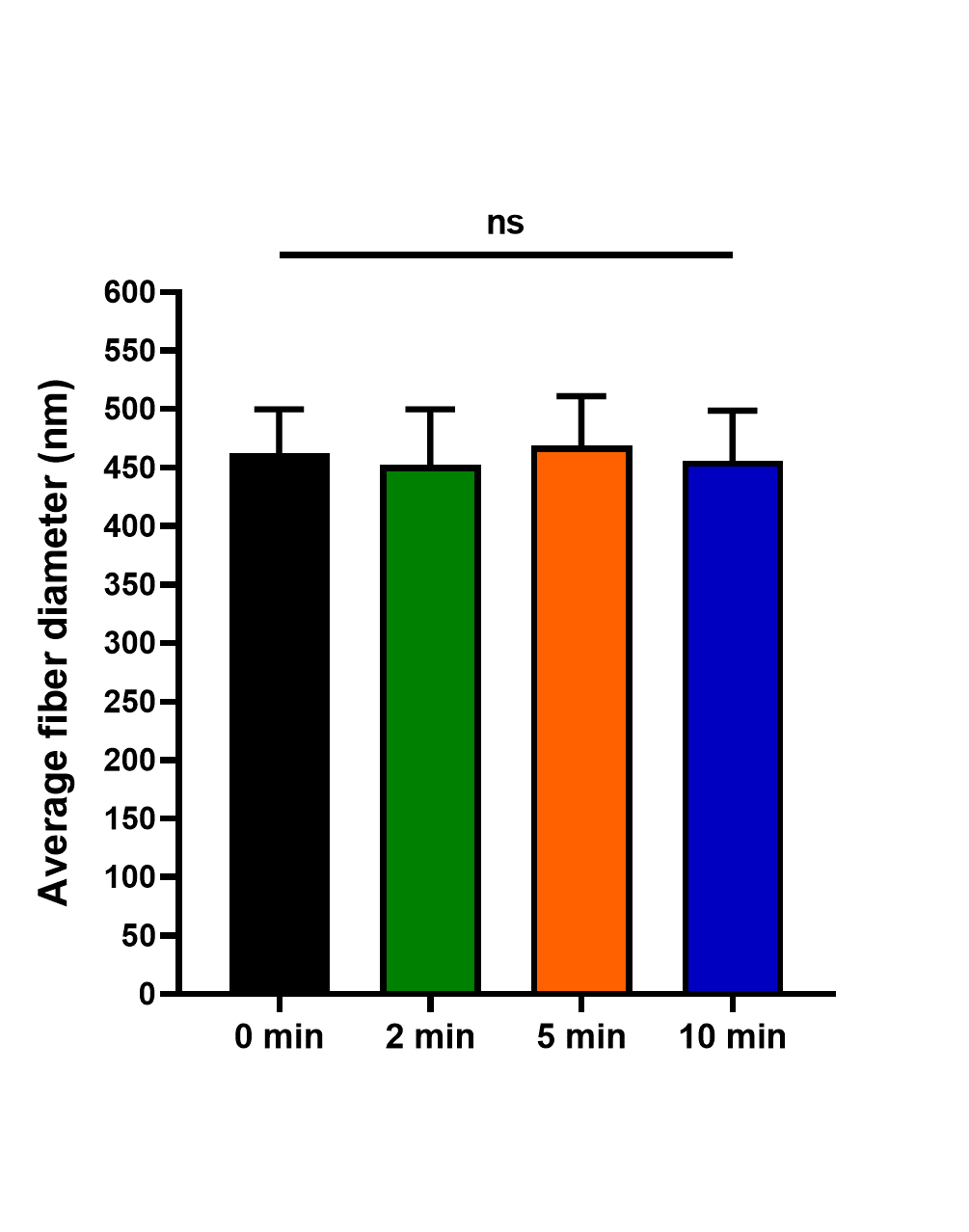


**Supplementary Figure 1.** Average fiber diameter of SF nonwovens with different water vapor annealing time points.


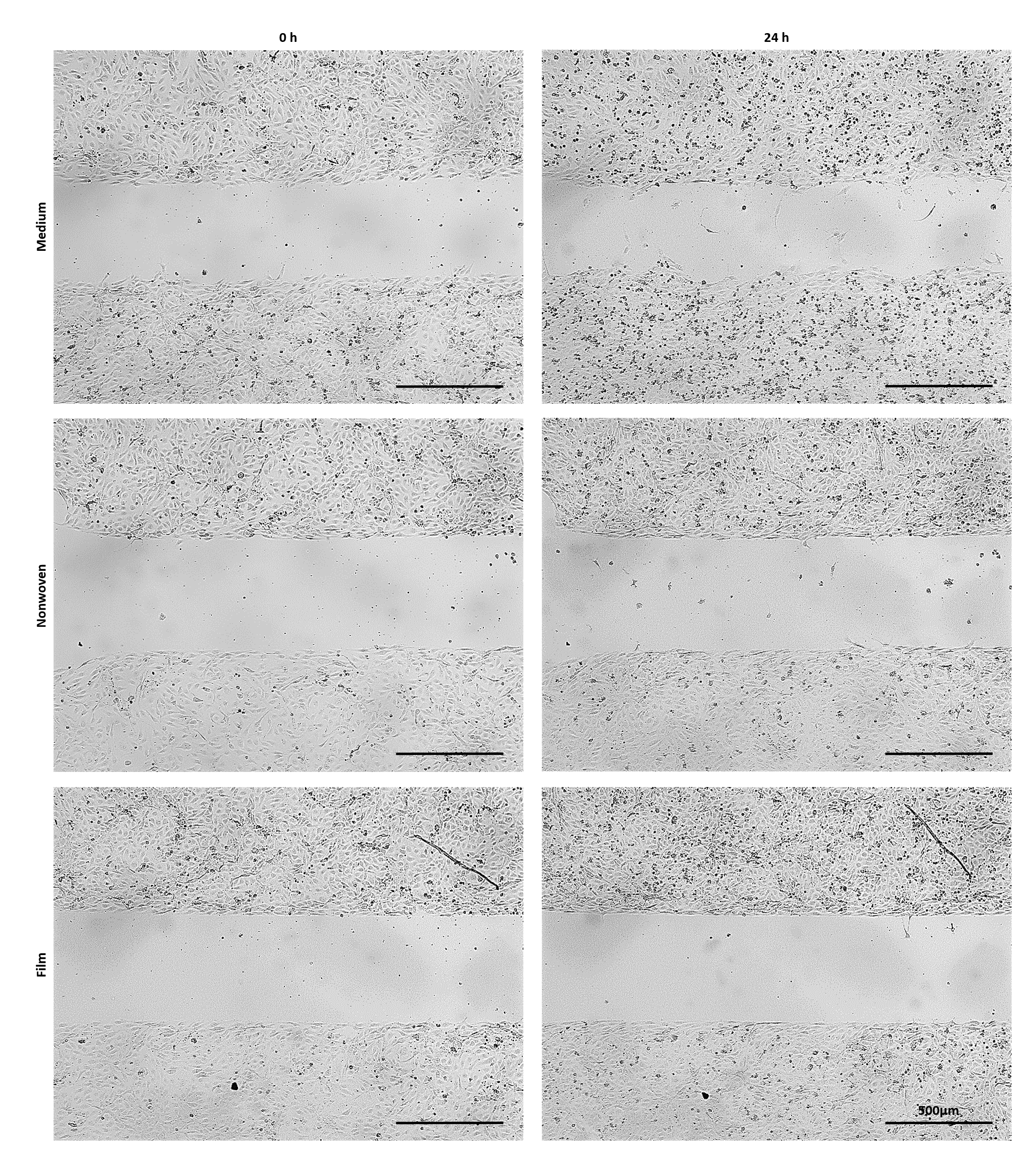


**Supplementary Figure 2.** Wound healing assay with HUVECs incubated with only medium, Nonwoven, and Film at 0 and 24 h.
